# Does the Dysregulation of Social Rhythms Syndrome (DYMERS) be Considered an Essential Component of Panic Disorders?

**DOI:** 10.2174/0117450179293272240328053722

**Published:** 2024-04-19

**Authors:** Diego Primavera, Giulia Cossu, Sonia Marchegiani, Antonio Preti, Antonio Egidio Nardi

**Affiliations:** 1 Department of Medical Sciences and Public Health, University of Cagliari, Italy Section of Psychiatry, Cagliari, Italy; 2 Department of Mental Health, ASL Medio Campidano, Cagliari, Italy; 3 Department of Neuroscience “Rita Levi Montalcini”, University of Turin, Turin, Italy; 4 Institute of Psychiatry, Federal University of Rio de Janeiro, Rio de Janeiro Brasil

**Keywords:** Panic disorders, Bipolar disorder, Social rhythms, New syndrome depressive episode, Mood disorder, DYMERS

## Abstract

This editorial explores the role of hyperactivity and social rhythm dysregulation in bipolar disorder (BD) and related syndromes. Social Rhythm Dysregulation Syndrome (DYMERS) is proposed as a common vulnerability across various disorders, including panic disorder (PD), attention deficit hyperactivity disorder, and post-traumatic stress disorder. A study conducted on a sample of elderly individuals participating in an active aging study investigated whether individuals with PD exhibit higher biological rhythm dysregulation compared to those without PD.

The sample, consisting of 119 individuals, revealed that those with a lifetime PD diagnosis scored significantly higher on the dysregulation of biological rhythms scale compared to those without panic disorder. A higher prevalence of depressive episodes was found in individuals with PD at the time of the interview. Notably, a small sample of elderly individuals with panic disorder, voluntarily selected for a physical exercise trial, showed a significantly higher level of dysregulation of social rhythms compared to those without panic disorder.

This study opens a debate on the accuracy of paper and pencil screening tests for bipolar disorders, especially regarding false positives in individuals with panic disorder. Our hypothesis is that DYMERS could be a shared vulnerability substrate for various disorders, serving as a basis for bipolar onset in the presence of a hyperactivity profile, even with genetic features. The data collected from older adults suggest that social rhythm dysregulation is a typical feature of PD, regardless of the coexistence of a depressive episode.

While the study has limitations due to a small sample size, the findings warrant careful analysis and suggest the need for larger-scale replication studies. If confirmed, the dysregulation of rhythms and its association with depressive disorders highlight a significant area of vulnerability for serious psychopathological disorders, emphasizing the importance of extending research to younger populations.

## 1 INTRODUCTION

Mental disorders, mainly Bipolar Disorder (BD), are linked to an alteration of metabolic pathways. These can be disrupted in various ways, including the dysregulation of biorhythms and sleep. Sleep disruption plays a central role in the onset, recurrence, dysfunction, and unfavorable health outcomes of various mental disorders, with particular emphasis on bipolar disorder (BD). External environmental elements, such as artificial light and road traffic noise, contribute to sleep disturbances, deeply influencing immune-hormonal circadian timing mecha-nisms (24-hour rhythms) and other intrinsic rhythms that have undergone an evolutionary process to optimize human behavior, synchronizing it with circadian rhythms concerning light variations and other environmental fac-tors like weather and seasons [1, 2].

A hypothesis has been advanced, asserting that among the triggering factors for the onset of bipolar disorder, there are alterations in circadian rhythms [3]. The modern age has altered our lifestyle, potentially leading to a disruption in the sleep-wake cycle. Failure to rest at night might result in increased energy levels as an adaptation. However, this deviation from the energy expenditure pattern established over millennia of evolution creates a disparity between current habits and the evolutionary perspective [2].

Clinical studies in this field have burgeoned during the COVID-19 pandemic and subsequent lockdowns, introdu-cing the concept of the “Dysregulation of Mood, Energy, and Social Rhythms Syndrome” (DYMERS). The interest in this new syndrome stems from its seeming crucial role in the exacerbation of chronic conditions, representing a distinct clinical profile associated with stress. This underscores the importance of social rhythms in stress prevention. These investigations have unveiled the existence of DYMERS and its potential implications for health and well-being [4]. According to this perspective, Social Rhythm Dysregulation Syndrome (DYMERS) could be configured as a condition of vulnerability common to various syndromes, including panic disorder (PD), attention deficit hyperactivity disorder, post-traumatic stress disorder, and others [5]. A common characteristic of these disorders would be a condition of hyperactivation, at least episodic, not always sufficient to define an episode of hypomania, and therefore also without comorbid bipolar disorder [6]. However, such episodes are sufficient to test (false) positive in pencil and paper screening tests for bipolar disorders.

The aim of this work is to verify whether, in a sample of elderly individuals selected for a study on active aging, individuals with PD will be found to have a higher score on a biological rhythm measurement scale than people without PD. Individuals who did not have a lifetime diagnosis of BD were selected from the database built for a randomized controlled trial on the effect of physical activity on active aging [7]. Another preliminary exclusion criterion was the absence of psychotic disorders. The selected group was divided by whether they had a diagnosis of Lifetime PD (according to DSM-IV). The two groups were compared based on the Brief Symptom Rating Scale (BSRS) and Patient Health Questionnaire-9 (PHQ-9) scores. The BSRS [8] in Italian Version [9] is a ten-item tool assenting the (ir)regularity of daily activities (sleeping, eating and social contacts) during the last week. Each item was rated from 1 (maximum regularity) to 6 (maximum irregularity). The PHQ-9 in the Italian version [10] is a self-administered questionnaire, detecting each of the 9 core criteria of a major depressive episode; the scores of each item range from “0” (complete absence) to “3” (almost every day). The cut-off for mild depression was 4/5 [11].

A total of 119 people were selected. The males were 53 (44,6%), and the females were 66 (55.4%). The mean age was 72.26 (±4.72), of which 112 were without a lifetime diagnosis of PD (females were 71 (59.7%), aged 72,24±4.75) and 7 with a lifetime diagnosis of PD (females were 5 (71.4%), aged 72.57±4.86).

The results of our study are summarized in Table **1**. The sample of people with a lifetime diagnosis of PD showed a significantly higher score on the dysregulation of biological rhythms scale than the sample without PD (25.00±7.21 *versus* 18.61±6.89, p=0.0027) measured with BSRS. All 7 individuals with PD showed a score higher than the general mean of the 119 subjects in the sample (score range PD 20-42. general average in the 119 individuals was 19.21±6.9). Twenty people with PD were found to be positive for depressive episodes (measured with PHQ-9) at the time of the interview than 5 people without PD (71.4% PHQ9>4 *vs* 17.8%, OR=11.5, CI95% 2.08-63.5). This preliminary study found that a (small) sample of elderly people with panic disorder, not belonging to a clinical center but voluntarily selected for a trial on physical exercise, showed a level of dysregulation of social rhythms significantly higher than people without the panic disorder (the entire sample did not present bipolar disorders or psychotic syndromes). The small size of the sample examined did not allow a multivariate analysis to determine whether and to what extent the difference in rhythm dysregulation in PD was determined by co-morbidity with the depressive episode. However, it appeared that in people without panic disorder, those with a depressive episode did not have a statistically higher level of rhythm dysregulation than those without a depressive episode (21,26±8.89 DE *versus* 18.03±6.59, p=0.066), as mentioned in Table **2**.

The results of this study, although preliminary and from a small sample, deserve careful analysis. It is known that panic disorder has frequent co-morbidity with bipolar disorder [12]. Panic disorder is one of the diagnoses that was found frequently in people who tested (false) positive on the paper and pencil screening in the absence of bipolar disorder [13], thus starting the debate on two opposing hypotheses, namely that the paper and pencil screening tests were not accurate [14] and that these instruments identified the entire spectrum of bipolar disorders far below what was (erroneously) believed to be the clinical threshold [15-18].

As an alternative to both of the two previous hypotheses, our group has advanced a third that the DYMERS could be a substrate of vulnerability common to many disorders and that this substrate could provide the basis for a bipolar onset in the case of co-presence of a hyperactivity profile. Hyperactivity, also with genetic features [11], could have presented not only in a pathological form but also with maladaptive connotations [12, 13].

## CONCLUSION

According to this point of view, PD could be interpreted as a consequence of DYMERS condition, which can occur with or without BD. Our data collected in older adults, therefore, with a low probability of developing BD in later years, show that the dysregulation of social rhythms seems to be a typical feature of PD, regardless of the co-existence of a depressive episode. However, the high frequency of depressive episodes in people with PD suggests the need to extend this type of research also to samples of young adults. If the dysregulation of rhythms and the association with depressive disorders were confirmed, an area of serious vulnerability for further serious psychopathological disorders would be highlighted. The limitations of this study are related to the low sample size. However, the data suggest replicating the study in larger samples.

## Figures and Tables

**Table 1:** The results of the study diagnosis of PD.

-	BSRS Score	Kruskal-Wallis Test	PHQ9>4	Chi-square with Yates’s Correction	OR (CI 95%)
PD - (N=112)	18.61±6.89	H=4.875 P=0.027	5 (71.4%)	8.394 P=0.004	11.5 (2.08-63.5
PD + (N=7)	25.00±7.21	-	20 (17.8%)	-	-
Total (N=119)	19.21±6.9	-	25 (21.00%)	-	-

**Note: **People without Panic Disorder = PD- People with Panic Disorder = PD + Brief Symptom Rating Scale score= BSRS SCORE Patient Health Questionnaire-9 >4 = PHQ-9>4

**Table 2:** Statistical levels of rhythm dysregulation.

-	BSRS Score	ANOVA 1 Way (1;110 df)
PD- / DE- (N=92)	18.03±6.59	F=3.457 P=0.066
DE + / PD - (N=20)	21,26±8.89	-
Total (N=112)	18.61±6.89	-

**Note: **People without Panic Disorder or Depressive Episode= PD - / DE – People with Depressive Episode without Panic Disorder = DE + / PD – Brief Symptom Rating Scale score= BSRS SCORE

## Data Availability

The data supporting the findings of the article is available in ClinicalTrials.gov NCT03858114 registered on 28 February 2019.

## LIST OF ABBREVIATIONS

BD: Bipolar Disorder
PD: Panic Disorder
BSRS: Brief Symptom Rating Scale
